# Future prospects for CD8^+^ regulatory T cells in immune tolerance

**DOI:** 10.1111/imr.12812

**Published:** 2019-10-08

**Authors:** Léa Flippe, Séverine Bézie, Ignacio Anegon, Carole Guillonneau

**Affiliations:** ^1^ Centre de Recherche en Transplantation et Immunologie UMR 1064 INSERM Université de Nantes Nantes France; ^2^ Institut de Transplantation Urologie Néphrologie (ITUN) CHU Nantes Nantes France; ^3^ LabEx IGO “Immunotherapy, Graft, Oncology” Nantes France

**Keywords:** CD8^+^ Treg, therapy, tolerance

## Abstract

CD8^+^ Tregs have been long described and significant progresses have been made about their phenotype, their functional mechanisms, and their suppressive ability compared to conventional CD4^+^ Tregs. They are now at the dawn of their clinical use. In this review, we will summarize their phenotypic characteristics, their mechanisms of action, the similarities, differences and synergies between CD8^+^ and CD4^+^ Tregs, and we will discuss the biology, development and induction of CD8^+^ Tregs, their manufacturing for clinical use, considering open questions/uncertainties and future technically accessible improvements notably through genetic modifications.

## 1 INTRODUCTION

CD8^+^ Tregs were the first suppressive cells reported but studies were then focused on CD4^+^ Tregs.^1^ Over the last two decades, we, along with others, deeply investigated the CD8^+^ Tregs.^2^ To date, significant progress have been made about their phenotype, their functional mechanisms, and their suppressive ability compared to conventional CD4^+^ Tregs.^3^ Through this in‐depth fundamental research, it has been learned more about their origin and development and such advances will lead to improvement in their generation including from stem cells (unpublished work discussed in this review).

Cell therapy has developed in recent years in the field of transplantation and autoimmune diseases with promising results. Current clinical trials use T cells and non‐T cells, and among Tregs, FOXP3^+^ and FOXP3^−^ CD4^+^ Tregs (phase I ONE, ALT‐TEN, phase I/IIa CATS1), but not CD8^+^ Tregs. We are at the dawn of the first in human clinical trial using CD8^+^ Treg‐cell therapy in transplanted patients (https://www.reshape-h2020.eu/). Experience from previous and current clinical trials using T cell therapy, combined with our extensive knowledge of CD8^+^ Tregs, incremented by the technical revolution of T Cell Receptor (TCR) and chimeric antigen receptor (CAR) engineering, in synergy with Treg promoting drugs, led us to the Eight‐Treg first in human clinical trial.

In this review, we will discuss the biology and development of CD8^+^ Tregs, their contribution to the tolerance status apart from CD4^+^ Tregs, their manufacturing for clinical use, considering open questions/uncertainties and future technically accessible betterments notably through genetic modifications.

## 2 MAIN CHARACTERISTICS OF CD8^+^ REGULATORY T CELLS

Despite a streamline work from several groups, the definition of CD8^+^ Tregs remains the major problem of this cell subset population and the reason for them not being assessed in clinical protocols. This lack in assessment is making marker discovery even more problematic and slow, leading to a vicious circle. The fact that, at steady state, expression of FOXP3, a sensitive marker of Treg, is lower in the CD8^+^ T cell population compared to the CD4^+^ T cell population in mouse, rat and human,^3^ has contributed to this disregard of CD8^+^ Treg's potential and interest, despite in vitro and in vivo data demonstrating their suppressive capacity. In addition, the methylation status of FOXP3 in human CD8^+^ Tregs showed an intermediate level of demethylation compared to one of the CD4^+^ Tregs for some locus of the FOXP3 gene.^3^ We showed that following ex vivo stimulation, human CD8^+^ Tregs sorted based on the expression of CD45RC, a splicing variant of the CD45 molecule key in the signaling of T cells that we use as a marker to isolate CD8^+^ Tregs^4^, ^5^, ^6^; FOXP3 is expressed by all expanded CD8^+^ Tregs that display highly upregulated levels of expression vs. blood unstimulated CD8^+^FOXP3^+^, suggesting that FOXP3 can correlate with the in vivo and in vitro suppressive potential of human CD8^+^ Tregs.^3^ In contrast, in a rat model of transplantation tolerance following costimulation blockade inducing in vivo CD8^+^ Tregs, FOXP3 was not upregulated,^4^ nor was it in a model of tolerance in rat using donor antigen therapy.^7^ FOXP3 is also not found particularly expressed in mouse CD8^+^ Tregs, and rather the transcription factor HELIOS or the surface marker CD122^+^ or CD28^‐^ are used to identify CD8^+^ Tregs in mice.^8^, ^9^ Other surface markers were also suggested and among the most frequently used CD25, CD127, GITR, CD39, Lag3, and CTLA‐4 that are used in different contexts and different species to identify CD8^+^ Tregs and analyze their suppressive activity in vitro and in vivo in autoimmune diseases, immune reactions to non‐self, cancer and materno‐fetal tolerance.^2^, ^10^ Indeed, using a combination of these markers to identify CD8^+^ Tregs, their suppressive action has been demonstrated in autoimmune diseases such as in the experimental autoimmune encephalomyelitis model,^11^, ^12^, ^13^ multiple sclerosis,^14^, ^15^, ^16^ systemic lupus erythematosus (SLE)^17^, ^18^ and primary biliary cirrhosis,^19^ in infection in humans with mycobacteria, human immunodeficiency virus or epstein‐barr virus,^20^, ^21^, ^22^, ^23^ in cancer in humans and mice^24^, ^25^, ^26^ or in transplantation in humans, mice and rats.^3^, ^4^, ^27^, ^28^, ^29^, ^30^, ^31^, ^32^ CXCR5 was used to identify antibody‐suppressor FOXP3^−^CD8^+^ cells in mice and essential in their function of suppression of humoral immunity, reducing germinal center B cells and CD4^+^ T follicular helper cells in the context of hepatocyte transplantation.^33^

A very recent study of single cell RNA sequencing on CD4^+^ Tregs has highlighted the importance of some transcription factors and extra‐membrane molecules.^34^ This work showed that CD4^+^ Tregs and CD4^+^ Tconvs can be distinguished by the ubiquitous expression of FOXP3, IKZF2 (IKAROS), TNFR2, IL2RA and IL2RB in mice and humans. Analysis using DGE‐RNA sequencing of fresh and activated CD8^+^CD45RC^low/‐^ Tregs allowed us to show the increase in FOXP3 expression and suppressive molecules after activation. However, nowadays no single cell RNA sequencing study is being published on CD8^+^ Tregs. Our analysis of single cell RNA sequencing data on fresh CD8^+^CD45RC^low/‐^ cells from healthy volunteers confirms the expression of molecules known in CD4^+^ Tregs such as TNFR2 but never clearly showed before in CD8^+^ Tregs (unpublished data).

**Figure 1:**
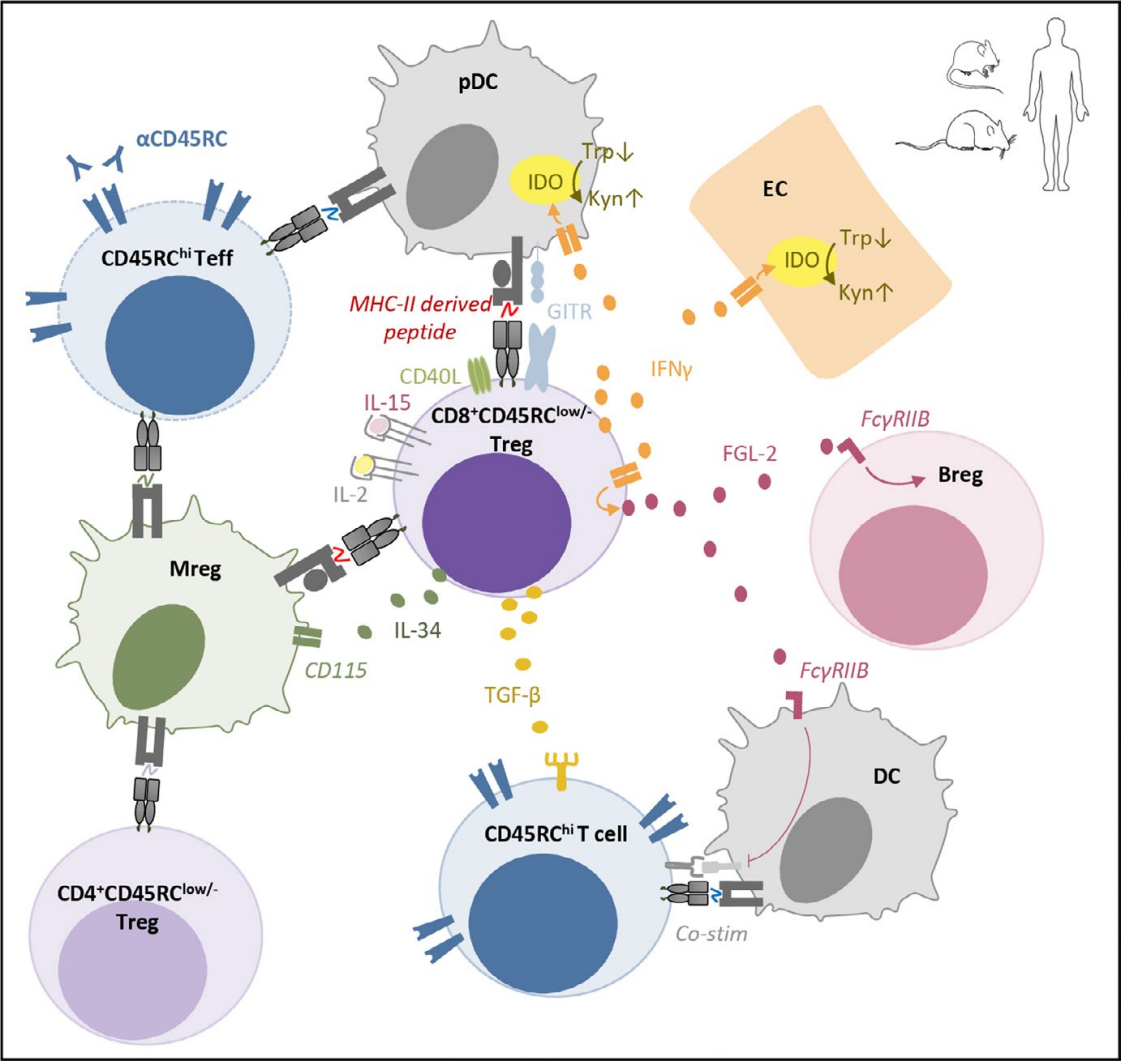
Schematic depicting identified mechanisms of action and markers of rat, mouse and human CD8^+^CD45RC^low/-^ Tregs. Breg, regulatory B cell; Co‐stim, costimulatory molecules; DC, dendritic cell; EC, endothelial cell; IDO, indoleamine 2,3‐dioxygenase; Kyn, kynurenin; Mreg, regulatory macrophages; pDC, plasmacytoid dendritic cell; Trp, tryptophan. Bended arrows indicate conversion or induction. Up and down arrows indicate increase and decrease, respectively

Soluble factors can also be used to identify and even sort CD8^+^ Tregs since the development of kit using bispecific antibodies allows the sorting of live cytokine‐secreting cells. We have shown that sorted human IFNγ^+^IL‐10^+^CD8^+^CD45RC^low/‐^ Tregs were more potent suppressor cells than the rest of the CD8^+^CD45RC^low/‐^ Tregs^3^ and this was also indicative of the role of these cytokines in CD8^+^ Treg suppressive activity, in particular IFNγ (Figure 1). Our interest in IFNγ came from the observations that blocking its activity abrogated the CD8^+^CD45RC^low/‐^ Treg suppressive activity in a model of allogeneic cardiac transplantation.^4^ We further demonstrated that indeed, IFNγ induced indoleamine 2,3‐dioxygenase (IDO) production by endothelial cells (ECs) of the graft and plasmacytoid dendritic cells, an enzyme catabolizing tryptophan essential for effector T cells proliferation.^4^, ^35^ IFNγ was also involved in fibroleukin‐2 (FGL2) induction, a cytokine acting further through regulatory B cell induction.^36^ We have also shown that IL‐34, a recently discovered cytokine involved in monocyte/macrophage differentiation, was secreted by approximately half of FOXP3^+^CD8^+^ and CD4^+^ Tregs.^37^ We showed that IL‐34 was involved in FOXP3^+^ Treg suppressive activity and acted as a suppressive cytokine since administration in a model of allogeneic cardiac transplantation in combination with a suboptimal dose of rapamycin‐induced transplant tolerance. This was the first description of a role for this cytokine in T cell biology and transplantation.^38^ Thus, a complex regulatory network of cells around CD8^+^ Tregs is acting to sustain tolerance.^39^ Other more classical cytokines such as TGFβ have also been described as playing a role in CD8^+^ Treg suppressive activity.^2^

Some of these molecules or mechanisms of action have been evidenced by study of the fetal‐maternal interface.^40^, ^41^ Indeed, in maternal tolerance where the allogeneic fetal trophoblast invades maternal tissues and interacts with maternal leukocytes, the immune system needs to be regulated to tolerate the presence of the fetus. CD8^+^ T cells are the main component of decidual T cells and CD8^+^ Tregs have been evidenced as important in the maintenance of a normal pregnancy.^42^, ^43^, ^44^ Decreased number and altered function of Tim‐3^+^CTLA‐4^+^CD8^+^ T cells correlated with miscarriage and blockade of Tim‐3 and CTLA‐4 inhibited production of anti‐inflammatory cytokines and were detrimental in the maintenance of the pregnancy.^42^ Allogeneic fetal trophoblasts express the non‐classical major histocompatibility complex (MHC) class I molecule human leukocyte antigen G which is needed for immune tolerance establishment and was shown to increase the number of Tregs.^45^, ^46^

MHC restriction preference of CD8^+^ Tregs has been shown as important for their suppressive activity and for some subpopulations of CD8^+^ Tregs different from what has been described for CD8^+^ Teff cells. Particularly in mice and humans, the best described population of mouse CD8αα^+^ TCRαβ^+^ Tregs or human CD8^+^ Tregs (no specific phenotype associated except pMHC restriction) has been described to preferentially recognize non‐classical MHC class I molecules Qa‐1 or HLA‐E that are orthologous genes.^47^, ^48^, ^49^, ^50^ These non‐classical MHC class I restricted populations have the property to recognize TCR, MHC or heat shock protein derived peptides (ie Qdm, HSP60sp) presented by Qa‐1 or HLA‐E.^50^, ^51^ We have also shown that this capacity to recognize MHC‐derived peptide is not restricted to non‐classical MHC class I molecule but also true for classical MHC class I molecule. We showed that peptides derived from a polymorphic region of the β1domain of MHC class II molecules from the donor and presented by recipient classical MHC I molecules can expand CD8^+^CD45RC^low/‐^ Tregs and induce tolerance in transplantation in an antigen‐specific manner in rats^7^, ^52^ and humans (unpublished data).

In this context, the identification of new membrane markers for CD8^+^ Treg populations to better define a consensus phenotype is a major challenge and objective for the future. In addition, a better understanding of the mechanisms involved in their development and function would allow to better understand and optimize their clinical potential.

## 3 SIMILARITIES, DIFFERENCES AND SYNERGIES BETWEEN CD8^+ ^AND CD4^+^ TREGS

A major aspect that remains to be properly addressed given the lack of assessment of CD8^+^ Tregs in diseases is the similarities, differences and synergies that could exist between CD8^+^ and CD4^+^ Tregs that could emphasize the interest in CD8^+^ Tregs (Table 1).

**Table 1:** Differences between CD8^+^ and CD4^+^ Tregs

	CD8^+^ Treg	CD4^+^ Treg
MHC	Class I, classical^7^ and non‐classical^47^	Class II^53^
Composition	Mostly memory^3^	Naive and memory^213^
Overall suppressive capacity	CD8> or =CD4^3^, ^32^, ^54^	
Suppression of memory and/or naive T cell responses	Memory and naive^58^	Naive^57^
Interferon gamma mediated suppression	Repeatedly described^3^, ^4^, ^65^, ^73^, ^74^, ^75^, ^76^, ^77^	Rarely described^70^, ^78^
IL‐2 induced proliferation	CD8> or =CD4^32^, ^103^, ^104^, ^105^, ^106^, ^107^	
IL‐15 induced proliferation	CD8> or =CD4^3^, ^54^, ^61^, ^111^, ^112^, ^113^, ^114^	
Preferred polyclonal stimulation	mAbs^3^	Beads^146^, ^147^, ^148^, ^149^

### 3.1 Function and mechanisms of action

A very obvious and important difference is that CD8^+^ and CD4^+^ Tregs recognize their cognate antigens presented by MHC‐I or MHC‐II molecules, respectively. Therefore, CD8^+^ Tregs will activate their suppressive activity on virtually all cells whereas CD4^+^ Tregs activation will occur only on cells expressing MHC‐II molecules. In the case of solid organ transplantation, CD8^+^ Tregs will activate on not only all donor graft cells but also on dendritic cells through indirect donor alloantigen presentation on recipient MHC‐I molecules.^7^ Thus, for both CD8^+^ and CD4^+^ Tregs, bystander inhibition of T and B cell activation will occur through antigen presenting cells (APCs) which express MHC‐I^+^ and MHC‐II^+^ molecules. In inflamed tissues non‐immune cells upon activation by IFNγ and/or TNFα may express MHC‐II molecules, but MHC‐I expression is constitutive by virtually all cells and its expression is also upregulated by IFNγ and/or TNFα. Thus, CD8^+^ Tregs will be advantageous, particularly in organ transplantation or graft versus host disease (GVHD) since suppression will additionally occur in all allogeneic cells, including ECs of vascularized grafts or host organs in GVHD. Another potential area in which MHC‐I antigen recognition might be useful is in the case of using in the future allogeneic off‐the shelf Tregs. Recipient cells attacking allogeneic CD8^+^ Tregs may be suppressed through MHC‐I recognition whereas CD4^+^ Tregs could only do it if the recipient cells express MHC‐II molecules, which can be the case for human T cells but this demands high levels of IFNγ‐mediated activation. Although a group recently published the structure of an induced CD4^+^ Tregs' TCR binding a pMHC,^53^ little is known about CD4^+^ Tregs pMHC restriction in contrast to CD8^+^ Tregs. This is probably due to the easiness to generate MHC class I/peptide tetramers that led to the opportunity to better identify and understand the TCR constraints and antigen recognition of CD8^+^ Tregs.

A direct comparison of CD8^+^ and CD4^+^ Tregs' overall suppressive capacity has not been done in many settings but in solid organ transplant^54^ and GVHD^32^ models in mice, suppression by CD8^+^ Tregs was superior to the one of CD4^+^ Tregs. We have observed that fresh, in vitro expanded human polyclonal and donor‐specific CAR engineered CD8^+^ Tregs were all superior to their CD4^+^ Treg counterparts (unpublished data and Ref. 3). Importantly, in certain tolerance models one or the other or both Treg subsets can be induced and play a role. As examples, in the same rat heart allotransplantation tolerance model, CD8^+^ and/or CD4^+^ Tregs were differently involved depending on the treatment used: blocking CD40‐CD40L induced tolerance that was entirely dependent and transferable by CD8^+^ Tregs,^4^ whereas donor‐specific blood transfusion as well as anti‐CD45RC MAb or IL‐34 administration tolerance was dependent on both CD8^+^ and CD4^+^ Tregs.^37^, ^55^ In these two last models adoptive transfer of each of the CD8^+^ or CD4^+^ Treg subsets resulted in tolerance in a fraction of the recipients that was lower than the one obtained when all Tregs were transferred.^37^, ^55^ Similarly, in a mouse model, GVHD was most efficiently inhibited when both CD4^+^ and CD8^+^ Tregs were used associated and the presence of CD8^+^ Tregs preserved a Teff‐mediated graft versus leukemia effect that was lost when CD4^+^ Treg cells were used alone.^56^ Interestingly in the perspective of simultaneously using CD8^+^ and CD4^+^ Tregs, both Treg subsets could be produced in vitro at the same time, such as when they are induced in the presence of APCs treated with IL‐34.^37^ Finally, the suppressive capacity of CD8^+^ and CD4^+^ Tregs to suppress naive and memory CD4^+^ T effector response has been compared in the transplantation field by different groups. This is a very clinically relevant situation to analyze the role of naive vs. memory CD4^+^ T effector responses, since both are activated at different time points during transplantation and memory T cell response is described as particularly difficult to control with usual immunotherapeutic or even immunosuppression protocols. Both naive and memory CD4^+^ Treg cells only suppressed naive and not memory CD4^+^ and CD8^+^ T responses.^57^ In contrast, CD8^+^ Treg cells suppressed both naive and memory effector CD4^+^ T cell responses.^58^

As far as mechanisms of action are concerned, several cytokines have been described as playing a role for both CD8^+^ and CD4^+^ Tregs, although not necessarily in all models or in vivo or in vitro situations. As an example, IL‐10‐mediated suppression has been described for CD8^+^CD122^+^ Tregs in mice 54 but not for human CD8^+^CD45RC^low/−^ Tregs.^3^

Similarly, TGFβ has been implicated in the suppressive function of some CD4^+^ Tregs and at least some types of CD8^+^ Tregs, such as mouse peptide‐specific CD8^+^ iTreg cells,^59^ CD8^+^FOXP3^+^CD25^+^ Tregs induced by corneal ECs^60^ and CD8^+^CD45RC^low/‐^ human Treg cells^3^ but not of CD8^+^CD45RA^+^CCR7^+^FOXP3^+^ Treg cells.^61^

IL‐34 is a cytokine with immunoregulatory properties by at least inducing M2 macrophages^38^ and that is expressed by both CD8^+^ and CD4^+^ Tregs and is able to prolong heart allograft survival.^37^

FGL2 immunoregulatory properties were first described in CD4^+^ Treg cells,^62^ FGL2 expression was then reported in mouse CD8αα Treg cells^63^ and in rat CD40Ig‐induced CD8^+^CD45RC^low/‐^ Treg cells functions. FGL2 function is at least mediated through the FcγRIIB receptor inhibiting dendritic cell maturation, inducing B cell apoptosis^64^ and generating regulatory B cells through unknown mechanisms.^36^ IFNγ is a mechanism of suppression not only of CD8^+^ Tregs^3^, ^65^, ^66^, ^67^ but also of CD4^+^ Tregs.^68^, ^69^, ^70^ The mechanism of suppression by IFNγ includes actions on endothelial and antigen presenting cells, such as induction of IDO and FGL2.

IL‐35 has been previously described as expressed by CD4^+^ Tregs^71^ but it has also been described as expressed and playing a suppressive role for tumor‐associated CD8^+^ Tregs.^66^, ^72^

Although IFNγ has proinflammatory consequences it has also been described as having pro‐tolerogenic actions, through the production of molecules such as IDO, iNOs and FGL2.^68^ IFNγ has been described as produced by several CD8^+^ Treg types 3, 4, 65, 73, 74, 75, 76, 77 although the function of IFNγ in CD8^+^FOXP3^+^ cells has not been specifically analyzed. In contrast, and although suppression through IFNγ has been described by CD4^+^ Tregs in certain models^70^, ^78^ CD4^+^FOXP3^+^ Tregs in general produce low levels of IFNγ.

PD‐1 has been described in mice to be expressed at lower levels in CD8^+^ Tregs vs CD4^+^ Tregs^32^ but PD‐1 expression is important for CD8^+^ Treg function,^79^, ^80^ as it is for CD4^+^ Tregs.^81^, ^82^ Additionally, PD‐L1 expressed by CD4^+^ Tregs^83^ and by human CD8^+^ Tregs^84^ suppress CD4^+^ Teff responses directly through PD‐1 or also through PD‐L1 expressed by DC and macrophages. Therefore, the use of PD‐L1‐Fc or agonistic anti‐PD‐1 antibodies would inhibit immune responses by both promoting Treg activity and by inhibiting Teff responses directly, as well as by inducing tolerogenic APCs.

Although cytotoxicity could be expected to be a widespread mechanism of suppression by CD8^+^ Treg cells and not of CD4^+^ Treg cells, only in some cases CD8^+^ Tregs have been described as cytotoxic 85, 86, 87, 88 and CD4^+^ Tregs have also been shown in some instances as cytotoxic,^89^, ^90^, ^91^, ^92^, ^93^, ^94^ thus this mechanism is shared by both types of Tregs.

### 3.2 Metabolic activity

Mechanistic target of rapamycin (mTOR) through mTOR complex 1 (mTORC1) and mTORC2 regulates activation and proliferation through the phosphorylation of several transcription factors.^95^ Mechanistic target of rapamycin signaling is high and increases the inflammatory response in Teff cells maintaining glycolysis whereas mTOR activity is low in CD4^+^ Tregs.^96^ Inhibitors of mTOR such as rapamycin directly bind and inhibit mTORC1. Activity of mTORC1 is necessary for CD4^+^ Treg cell function to maintain their metabolic status through lipogenesis.^97^ Therefore, rapamycin at moderate concentrations inhibits CD4^+^ Teff growth but promotes CD4^+^ Treg growth and function.^98^, ^99^ Although metabolic activity and mTOR function have not been directly analyzed in CD8^+^ Tregs, the metabolic status of memory CD8^+^ cells is similar to that of CD4^+^ Tregs^100^, ^101^ and different types of human CD8^+^ Tregs having a memory phenotype have been shown to expand in the presence of rapamycin,^3^, ^102^ thus, inclusion of rapamycin in the in vitro expansion of clinically applicable human CD8^+^ Tregs as for CD4^+^ Tregs is a logical approach to increase safety.

### 3.3 Proliferation in vitro and in vivo

Freshly analyzed human and mouse CD8^+^FOXP3^+^ Tregs have been reported as IL2Rα/CD25^high^ and CD127^low^ as CD4^+^FOXP3^+^ Tregs^103^ whereas human CD8^+^CD45RC^low/‐^ have a more complex phenotype.^3^ Transgenic rats with green fluorescent protein under control of the FOXP3 promoter are also CD25^high^CD127^−^ (unpublished data). The other chains of the IL2R are expressed in a large proportion of fresh human CD8^+^FOXP3^+^ Tregs comparable to the ones of CD4^+^FOXP3^+^ Tregs (unpublished data). With this expression of the IL2R chains similar to those of CD4^+^FOXP3^+^ Tregs it is logical that low dose IL‐2 administration in vivo to mice and human healthy volunteers expanded CD8^+^FOXP3^+^ Tregs in both species and even in a higher proportion in humans (6.7 ± 4.3 fold) as compared to CD4^+^FOXP3^+^ Tregs (1.8 ± 0.7 fold). The suppression capacity after IL‐2 treatment was also higher in CD8^+^FOXP3^+^ Tregs vs. CD4^+^FOXP3^+^ Tregs.^103^ Administration of low dose IL‐2 in cynomolgus monkeys expanded both CD8^+^FOXP3^+^ and CD4^+^FOXP3^+^ Tregs 10‐fold and 15‐fold, respectively when compared to the baseline levels and the suppressive effect after IL‐2 treatment was comparable for CD8^+^ and CD4^+^ FOXP3^+^ Tregs.^104^ Similar increase in CD8^+^ and CD4^+^ Tregs (11 to 14‐fold) was observed when using an IL‐2 mutein molecule.^105^ Increase in CD8^+^FOXP3^+^ Tregs along with CD4^+^FOXP3^+^ Tregs was also observed in patients with hepatitis C virus‐induced vasculitis 106 and type 1 diabetic patients 107 treated with low doses of IL‐2. In a model of GVHD in mice, administration of IL‐2 antibody complexes and rapamycin was synergic to CD8^+^FOXP3^+^ Tregs increase up to 30‐fold whereas CD4^+^FOXP3^+^ Tregs increased 4‐fold.^32^ In other studies, in patients treated with low doses of IL‐2, CD8^+^ Tregs were not reported,^108^, ^109^ although only a few of them described extensive immunophenotyping.^109^

Although IL‐15 favors thymic development of CD4^+^ Tregs along with IL‐2 through the IL‐2Rβ/γc cytokine receptor complex,^110^ IL‐15Ra is not expressed by CD4^+^ Tregs and these cells respond to IL‐15 only when soluble IL‐15Ra binds IL‐15 and presents it in trans.^111^ This explains why adult CD4^+^ Tregs do not respond to IL‐15 in vitro while they do in vivo.^111^, ^112^ In contrast, IL‐15 is necessary for optimal CD8^+^ Tregs expansion in vivo^113^, ^114^ and in vitro.^3^, ^54^, ^61^

In vitro expansion of CD8^+^ Tregs in the same conditions used to expand CD4^+^ Tregs has shown lower suppression activity in some cases but this may be related to the use of culture conditions that are optimal for CD4^+^ and not CD8^+^ Tregs.^115^ In this regard, we have observed that the use of beads coated with anti‐CD3 and anti‐CD28 MAbs ideal for the culture of CD4^+^ Tregs is toxic for human CD8^+^ Tregs while being well‐tolerated by mouse CD8^+^ Tregs (manuscript in preparation). It is then important when comparing in vitro cultured CD8^+^ and CD4^+^ Tregs to use the best culture conditions for each cell type.

## 4 BIOLOGY AND DEVELOPMENT OF CD8^+^ TREGS

### 4.1 Origin

The thymic origin of CD8^+^ nTregs is however still poorly described. In 2016, the presence of CD8^+^CD28^low^ Treg cells in peripheral blood mononuclear cells (PBMCs) and in children's thymuses was described and also their thymic origin demonstrated.^9^, ^116^ CD8^+^ Tregs are associated with different phenotypes depending on the studies (CD122, CD28, CD45RC, CD103, PD‐1) but mostly their phenotype is rather associated to a differentiation status of central memory cells or effectors memory as characterized by the absence of CD28, CD62L or CD122 expression.^3^, ^117^ Other teams describe them rather through the expression of CD44, CCR7 and CD62L which are markers of central memory cells 3, 73 or CD122^+^ and CD28^+^.^32^, ^54^ When activated, CD8^+^ Tregs lose the expression of the CD45RA, distinguishing the naive state, to express CD45RO, a marker of memory state, and are dependent on IL‐2 and IL‐15. Naive CD8^+^ Tregs leave the thymus and such natural CD8^+^ Tregs have been shown by us with a limited suppressive capacity in both rat and human.^3^, ^4^ Indeed, adoptively transferred naive rat CD8^+^CD45RC^low/‐^ Tregs cannot inhibit acute transplant rejection in a fully allogeneic model of transplantation^4^ and human CD8^+^CD45RC^low/‐^ Tregs from blood of healthy individuals are more potent suppressor cells in vitro following anti‐CD3 and anti‐CD28 stimulation.^3^ Effector and memory Tregs have a much more potent suppressive capacity than naive Tregs in mice and humans.^118^ Effector Tregs are found in the blood and in secondary lymphoid organs, while memory Tregs are found mainly in peripheral tissues and in secondary lymphoid organs.

Transcription factors can identify the thymic origin of CD8^+^ Tregs. Some were suggested for a long time as reliable markers of Tregs from thymic origin. Indeed, HELIOS, a member of Ikaros family of zinc finger transcription factor, is expressed by 100% of CD4^+^FOXP3^+^ Tregs in the thymus and 70% of CD4^+^FOXP3^+^ Tregs in peripheral lymphoid tissues.^119^ HELIOS seems to be involved in the stabilization of the phenotype of CD4^+^ and CD8^+^ Tregs in an inflammatory context.^8^ In a mouse model deficient for HELIOS, CD8^+^ and CD4^+^ Tregs were not able to control effector T cells responses. This demonstrates HELIOS' involvement in suppressive functions, differentiation and survival of Tregs.^8^, ^120^ Human CD8^+^CD45RC^low/‐^ cells co‐express HELIOS and FOXP3.^3^ FOXP3 seems to be a key transcription factor in the development of Tregs including CD8^+^ Tregs since silencing FOXP3 with siRNA abrogated the ability of CD8^+^ Tregs to suppress anti‐DNA antibodies in a lupus model in mice.^121^ Although adoptive transfer of CD4^+^FOXP3^+^ Tregs can cure immune dysregulation, polyendocrinopathy, enteropathy, X‐linked syndrome in FOXP3‐deficient mice^122^ this does not exclude the possibility that transfer of CD8^+^FOXP3^+^ Tregs could also have a similar effect. This is an important experiment that has not been reported and would confirm a physiological role in immune homeostasis for CD8^+^FOXP3^+^ Treg. Induced CD8^+^ Tregs express less FOXP3 than CD4^+^ Tregs.^123^ However, we have shown a specific expression of FOXP3 in natural CD8^+^CD45RC^low/‐^ Tregs while we do not find expression in CD8^+^CD45RC^high^ effector T cells. This expression of FOXP3 is all the more important after cell activation.^3^ This expression is correlated with the expression of suppressive molecules such as CTLA‐4, GITR, IFNγ and TGFβ. In mice, other transcription factors have been described as involved in the development and function of CD4^+^ Tregs but they have not yet been described for CD8^+^ Tregs. IFR4 was shown essential for the expression of Blimp‐1 in the differentiation of CD4^+^ effector Tregs^124^; the canonical pathway nuclear factor‐kappa B is also involved in the development and function of CD4^+^ Tregs by performing inducible deletions of some subunits of the complex. The c‐Rel subunit was critical in thymic development, while the p65 subunit was essential for maturing Tregs and maintaining immune tolerance.^125^

### 4.2 Targeted therapies modulate or synergize with CD8^+^ Tregs

Tregs have been well described to be induced or synergize with antibody or cytokine therapies, to be the main effector regulatory mechanisms boosting the efficacy of such therapies and being key to long‐term sustained effect. Thus, new therapies should strategically aim to promote Tregs to induce a satisfactory control of immune responses. Strategies of costimulation blockade have been shown to efficiently induce and mediate their tolerogenic effect through CD8^+^ Tregs. Blockade of the CD40/CD40L costimulatory pathway is a well‐known strategy in transplantation for its potential in animal models in tolerance induction, although its translation to the clinic has been slowed down due to thromboembolic events.^126^ We have shown that blockade of the CD40/CD40L pathway using an adenovirus encoding CD40Ig, a chimeric molecule, in a model of fully incompatible cardiac allotransplantation in rats resulted in tolerance induction dependent on CD8^+^CD45RC^low/−^ Tregs.^2^, ^4^, ^35^, ^127^ Blockade of the 4‐1BB/4‐1BBL costimulatory pathway together with immunization in mice was also shown to induce antigen‐specific CD8^+^ Tregs acting in a IFNγ and TGFβ dependent manner.^59^ In this model IFNγ directly stimulates CD8^+^ Tregs to induce a TGFβ‐based suppression.^59^ In contradiction, 4‐1BB has been shown as important for the suppressive activity of CD8^+^ Tregs in a model of allergic inflammation.^128^ We and others showed that IFNγ and TGFβ were key mediators of the suppressive activity of CD8^+^ Tregs.^4^, ^68^, ^129^, ^130^ Blockade of ICOS/B7h costimulation also showed potent induction of alloantigen‐specific CD8^+^PD1^+^ Tregs in vivo in a model of heart grafts in mice.^131^ More surprisingly, targeting CD3, a molecule expressed by definition by Treg, has shown effectiveness in inducing both CD4^+^ and CD8^+^ Tregs in a collagen induced arthritis model of rheumatoid arthritis in mice.^132^, ^133^ In this model, CD8^+^ Tregs, unlike CD4^+^ Tregs, potently inhibited Th17 responses, thereby inhibiting a wider range of inflammatory pathways. The authors also showed that monocyte membrane bound TNFα increased FOXP3 expression in CD8^+^ T cells. Administration of hOKT3g1 (Ala‐Ala) in patients with type 1 diabetes mallitus halted disease progression for >1 year and was associated with increased CD8^+^ FOXP3^+^ Tregs.^134^ Induction of CD8^+^ Tregs was dependent on TNF and associated with TNFR2 expression by the CD8^+^ Tregs.^135^ There is a ying and yang action observed by several groups for cytokines such as TNFα and IFNγ. Anti‐TNF antibodies are used in Crohn's disease and have shown effectiveness in management of the disease but also common failure.^136^ The importance of action of these cytokines on and by CD8^+^ Tregs, but also more recently by CD4^+^ Tregs,^137^ should certainly be taken into consideration in the potential limited effect of cytokine‐targeting drugs in autoimmune diseases.

Co‐administration of CD8^+^ Treg therapy with costimulation blockade is also showing synergistic efficacy. In a model of skin transplantation in mice, CD8^+^CD122^+^PD‐1^+^ Tregs expanded ex vivo combined with costimulation blockade of CD40/CD154, but not of B7/CD28, synergizing to prolong the allograft survival in an IL‐10‐dependent manner.^113^ In fact, blockade of the B7/CD28 costimulation pathway has been also shown by us as being detrimental to CD8^+^ Treg‐mediated tolerance. Simultaneous blockade of the CD40/CD40L and CD28/B7 interactions using CD40Ig and anti‐CD28 mAbs abrogated in 50% of the recipients tolerance, inhibited CD8^+^ Treg induction and modified the regulatory mechanisms taking place in the remaining recipients that did not reject their allograft,^138^ suggesting that the B7/CD28 costimulation is required for CD8^+^ Treg expansion and function.

CD8^+^ Treg induction following administration of depleting, modulating or blocking drugs has been very poorly addressed. There are several strategies under investigation for their potential in modulating the Teff/Treg balance, that are not directly targeting costimulation blockade but rather eliminating Teff of activating Tregs. We have shown that modulation of the Teff/Treg balance using anti‐CD45RC mAbs depleting CD45RC^high^ cells, ie naive and TEMRA cells, but preserving CD45RC^low/−^ cells, ie Tregs, can efficiently induce transplant tolerance in rats and humanized immune mice.^55^ The mechanisms of tolerance involved the transient depletion of naive and TEMRA cells at the time of transplantation and during 10‐20 days associated with a transient increase of CD4^+^ and CD8^+^ Tregs. In this model we demonstrated that upon arrest of the treatment and return to normal level, CD4^+^ and CD8^+^ Tregs gained antigen specificity and were functionally superior as shown by in vitro suppressive assays, in vivo adoptive cell transfer in transplanted recipients and transcriptomic analysis. We have similar unpublished results in a model of GVHD in rats and nod scid gamma (NSG) mice and in a model of lupus (unpublished data). In a lupus model in mice, administration of T cell targeting nanoparticles loaded with IL‐2 and TGFβ significantly expanded CD4^+^ and CD8^+^ Tregs that could reduce the disease.^139^ Cytokine therapies such as the one using IL‐34 has been shown to also modulate the Treg/Teff balance by differentiating monocytes into regulatory macrophages that could in turn induce CD8^+^ and CD4^+^ Tregs responsible for the long‐term tolerogenic effect.^37^

## 5 CD8^+^ TREG CLINICAL TRIAL IN KIDNEY TRANSPLANTED PATIENTS

**Figure 2:**
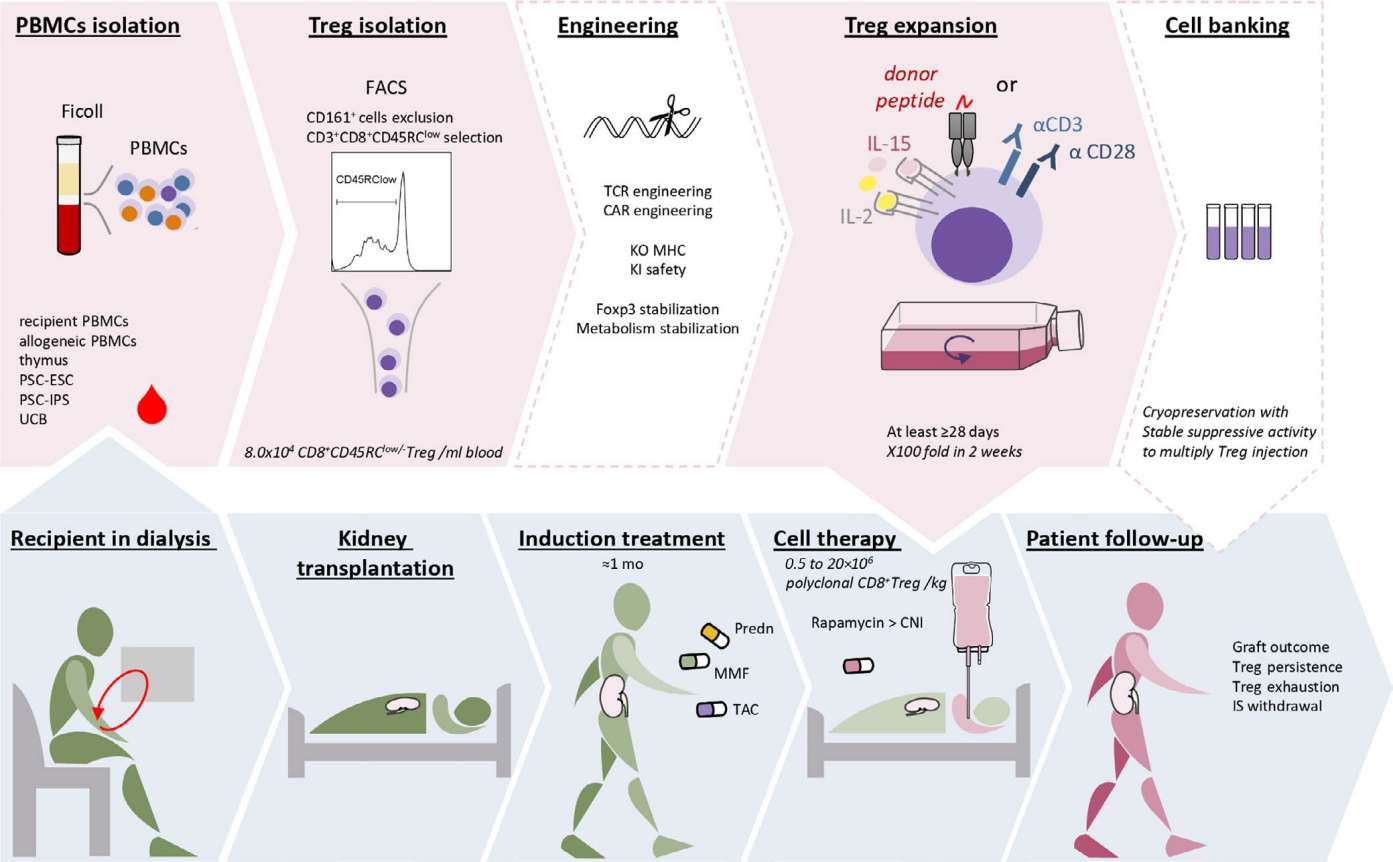
CD8^+^ Treg‐based therapy process. In vitro steps are indicated in pink background, optional steps in blank background and clinical steps in blue background. CNI, calcineurin inhibitor; KI, knock‐in; KO, knock‐out; MMF, mycophenolate mofetil; Prdn, prednisolone; TAC, tacrolimus; UCB, umbilical cord blood

While CD8^+^ T cells have been used in clinic as effector cells to treat cancer and infectious diseases (NCT00791037, NCT01325636, NCT01475058, NCT00110578), CD8^+^ Tregs have never been used as suppressive cells to treat autoimmune diseases or to control transplant rejection. Indeed, the lack of interest for CD8^+^ Tregs due to the difficulties of identification and characterization of these cells, in contrast with the extensive knowledge acquired for years on CD4^+^ Tregs, and the urgency for HSC‐grafted patients with fatal outcome supported rapid translation of CD4^+^ Treg to the clinic for cell‐based therapies.^1^ The new interest for CD8^+^ Tregs over the past two decades has strengthened their therapeutic potential, leading to a future first in human phase I CD8^+^ Treg‐cell therapy in kidney transplanted patients, named Eight‐Treg, supported by the ReSHAPE consortium, and that will take place in 2021^2^ (https://www.reshape-h2020.eu/) (Figure 2). The primary objective of the Eight‐Treg Phase I trial will be to determine the safety and dose of CD8^+^ Treg cells. CD8^+^ Tregs showed no cytotoxicity in vitro or in vivo in NSG mouse models and we observed comparable suppressive activity for CD8^+^ Tregs and CD4^+^ Tregs in vitro.^3^ Currently, the phase I clinical trials using CD4^+^ Tregs evaluate the cell toxicity by a dose escalation from 5 × 10^5^ to 2 × 10^7^ cells/kg in solid organ transplanted (SOT) patients and from 1 × 10^5^ to 3 × 10^7^ cells/kg in hematopoietic stem cell transplantation (HSCT) patients (NCT02371434 ONEnTreg13, NCT03444064, NCT01050764, NCT00725062, NCT03198234). In particular, the previous ONETreg1 clinical trial showed the safety of a 1 × 10^7^/kg CD4^+^ Tregs infusion in patients, and the downstream Two Study is underway to evaluate the efficacy of this dose in the treatment of kidney transplanted patients (NCT02129881, 2017‐001421‐41). Even the highly cytotoxic tumor infiltrating lymphocytes (TIL) CD8^+^ cells infused up to 3x10^9^ cells/kg in patients with melanoma did not induce serious adverse events (NCT01118091, NCT01236573).^140^ Altogether, these results suggest a low risk in receiving a high dose of CD8^+^ Tregs for patients. Therefore, the Eight‐Treg protocol is designed for the administration of escalating cell dose every three patients, from 3 × 10^5^ to 2 × 10^7^ CD8^+^ Tregs/kg in 12 kidney transplanted patients (https://www.reshape-h2020.eu).

The type and dose of immunosuppressive drug treatments are critical to ensure patient safety whether the cell therapy is not effective, but should not be deleterious to the adoptively transferred CD8^+^ Treg cells. We have recently shown that mycophenolic acid stopped their proliferation in contrast to rapamycin.^3^ In addition, we observed that rapamycin promoted CD8^+^ Treg proliferation and suppression capacities.^3^ Similarly, calcineurin inhibitor drugs (CNIs) were deleterious to the persistence of CD4^+^ Tregs in patients up to 1 year after kidney transplantation, while rapamycin monotherapy promoted CD4^+^ Tregs in SOT and HSCT patients^141^, ^142^, ^143^, ^144^ (NCT00803010). Nowadays, some clinical trials focus on the impact of different IS drugs, including rapamycin, on the frequency and function of CD4^+^ Tregs in SOT patients, and the inclusion of the analysis of CD8^+^ Tregs counterpart would be of great interest (NCT01014234, NCT01640743). In ongoing clinical trials in SOT, induction treatment is generally composed of tacrolimus + MMF+prednisolone for 1 month. Meanwhile, CD4^+^ Tregs are transferred adoptively rather after the critical rejection period to avoid any erroneous conclusion about any cytotoxic effect induced by the injected cells, between 1 week and 6 months after transplantation. Thus, ongoing clinical trials switch from tacrolimus to rapamycin one month after SOT to spare CD4^+^ Tregs at least and maybe to promote them (TASK, DelTa).^145^ This rational strategy will be applied to the Eight‐Treg clinical trial.

It is important to note that the Treg cells product, with or without genetic modifications, is classified as an Advanced Therapy Medicinal Product and regulated by the directive 1394/2007, aiming at standardizing the manufacturing process. The purity of cell product, the expression of key markers, and the cytotoxic and suppressive functions will be controlled. Fortunately, the manufacturing processes of CD4^+^ Tregs and CD8^+^ TILs used in ongoing clinical trials are helpful to set up the good manufacturing practices manufacturing of CD8^+^ Tregs product.^140^, ^146^, ^147^, ^148^, ^149^, ^150^ In addition, these processes can be upgraded by technical advances, such as clinical grade flow cytometry cell sorting. With this equipment, we can isolate 8.0 × 10^4^ CD8^+^ Tregs/mL blood, based on negative expression of CD161, low/neg expression of CD45RC and positive expression of CD8 marker from peripheral blood lymphocytes. Then, the cells can be highly expanded, about 200 fold in 2 weeks, while maintaining their suppressive capacity and without acquiring cytotoxic function.^3^ Although beads coated with anti‐CD3/anti‐CD28 mAbs are generally used to expand CD4^+^ Tregs, CD8^+^ Tregs are rather stimulated using the OKT3 clone, as CD8^+^ TIL for cell therapy in cancer, associated with CD28 stimulation using the CD28.2 mAb clone.^140^ Besides, many ongoing clinical trials are evaluating the therapeutic potential of antigen‐specific Tregs, such as donor alloantigen‐specific Tregs in SOT (DarTregs in the delta, NJLT001, DART, ARTEMIS, LITTMUS‐MGH) or tumor cells specific Teffs (TILs) in cancer (NCT01118091, NCT01236573, NCT03610490, NCT03068624), and TCR and CAR engineering is a promising alternative strategy for antigen‐specific cell therapy in transplantation and autoimmune diseases.^151^ Actually, we observed higher efficacy of donor MHC‐I‐specific CAR CD8^+^ Tregs than polyclonal ones in MHC‐I restricted transplantation models in NSG mice in which this MHC‐I antigen was incompatible (unpublished data). Our current manufacturing process would allow us to infuse up to 1 × 10^8^ polyclonal CD8^+^ Tregs/kg or 1 × 10^7^ CAR‐CD8^+^ Tregs/kg in patients.

An important problem for the success of Treg cell‐based therapies is the persistence of the infused cells in the patient. However, the lack of specific markers for CD8^+^ Tregs is a limitation for their tracking in blood and protocol biopsies of patients. Indeed, FOXP3 has been shown to be transiently overexpressed in Teffs when stimulated while there are technical problems in analyzing treg‐specific demethylated region methylations in biopsies.^152^, ^153^ There are also technical problems in detecting key cytokines to track IL‐34^+^IFNγ^+^TGFβ^+^CD8^+^ Tregs. Thus, new methods of systematic proteomic and transcriptomic analyses are emerging. Notably, deuterium is currently used in the TASK clinical trial (NCT02088931) to track adoptively transferred CD4^+^ Tregs in blood and in the grafted kidney, and this method might be considered to track CD8^+^ Tregs in patients.

Early exhaustion of adoptively transferred Tregs would affect their persistence and thus limit their therapeutic effect. Exhaustion of Tconv is reported as the expression of a combination of inhibitory receptors, activation and memory markers, such as PD‐1, TIM‐3, LAG‐3, CTLA‐4, CD160, TIGIT, BTLA‐4, and correlates with improved outcome of transplanted patients.^154^, ^155^ However, PD‐1 is not only a discriminant marker but also plays a functional role in the suppressive activity of both CD4^+^ and CD8^+^ Treg subsets,^84^ while the CTLA‐4 and LAG3 markers of human CD8^+^ Tregs may have a role.^27^, ^28^, ^156^, ^157^ It is important to note that the CD8^+^ Tregs used for the Eight‐Treg trial show no phenotypic sign of exhaustion after 2 weeks of culture, and survive and still proliferate for at least 1 month in vitro.^3^

Co‐treatment with IL‐2 is considered to promote the persistence of CD8^+^ Tregs in patients, as required for ex vivo culture.^3^, ^103^, ^104^, ^105^, ^106^, ^107^ Encouraging results were obtained in HSCT patients treated with low dose IL‐2, expanding a functional CD4^+^Foxp3^+^ Treg subset associated with a lower incidence of GVHD while maintaining a low viral infection incidence^158^, ^159^ (NCT00539695). However, soluble circulating IL2‐R can mediate sequestration of IL‐2 and limit its effect 160 (NCT01927120). Further investigations regarding the dose and duration of IL‐2 administration are required. Here again, rapamycin can promote the persistence and stability of CD8^+^ Tregs face to an inflammatory environment.^3^, ^161^

To cope with the lack of persistence of the infused Tregs, we could multiply the injections of CD8^+^ Tregs as in CD4^+^ Treg‐based clinical trials (RSMU‐001: NCT01446484, Treg: NCT01624077, NCT02749084). This process requires the production of a large amount of Tregs and reliable storage with a stable suppressive function.^162^ Long‐term culture and stable cryopreservation of CD8^+^ Tregs are possible.^3^

## 6 NEW SOURCES OF TREGS: DIFFERENTIATION FROM PLURIPOTENT STEM CELLS

Although cell therapy with Tregs is very promising for treating GVHD, rejection after transplantation and autoimmune diseases, one of the limitations is the source of cells to be injected. The cell source must be easily accessible and allow a large number of cells to be donated. PBMCs and cord blood cells are the most traditional sources. They also allow autologous treatments. However, the prevalence of Tregs in these sources is quite low and requires more rounds cell expansion.^163^

Recently a study has shown the possibility of using Tregs from the thymus of children recovered after cardiac surgery. Indeed, cardiac intervention in children routinely requires the removal of the thymus and these thymuses are then thrown away. However, there are more Tregs cells in a child's thymus than in an adult's entire blood volume and 100 times more than in a unit of cord blood, making it a very interesting source.^164^ Nevertheless, this source requires a very complex organization between surgical teams and teams of biologists to collect the thymus and extract the cells and the Treg product would be from allogeneic sources to the recipient.

Since 1998, human embryonic stem cell has been the ultimate source of cells for regenerative medicine. Thanks to their pluripotent characteristic, they can differentiate into any cell in the body and are able to renew themselves indefinitely. More recently, induced pluripotent stem cells (IPSC) were obtained by reprogramming of adult somatic cells using transduction of four factors essential for pluripotency in mice and then in humans.^165^, ^166^ This IPSC could lead to tailor‐made regenerative medicine. This avoids alloreactivity and therefore potentially reduces the doses of immunosuppressive treatment, which has many side effects. In recent years, studies on the differentiation of T cells from embryonic stem (ES) or induced pluripotent stem (iPS) cells have been carried out mainly in the field of oncology. In 2009, Timmermans et al were the first to demonstrate T cell differentiation from ES using an OP9 cell co‐culture protocol.^167^ They reported CD3 and TCR expression and IFNγ secretion after stimulation of their cells via TCR. Nowadays, several groups focus on obtaining T cells with antigenic specificity and have shown the possibility of reprogramming specific antigen T cells into IPSC: T‐IPS. During the differentiation of these T‐IPS the antigenic specificity is preserved.^168^, ^169^, ^170^, ^171^ All these studies showed the great potential of IPS‐derived T cells as an alternative source for T cell‐based immunotherapy in cancer treatments. This is why the use of ES‐ or IPSC‐derived Tregs is also considered with great interest in the treatment of GVHD or in autoimmune diseases. In 2012, R. Haque et al showed that the transduction of FOXP3 during the differentiation of IPSC into T cells led to the production of functional CD4^+^FOXP3^+^ Tregs in mice.^172^ Indeed, these CD4^+^FOXP3^+^ Tregs derived from IPSC were able to secrete suppressive molecules such as IL‐10 and TGFβ and they controlled autoimmunity in an arthritic mouse model. The same group showed in 2016 the use of Tregs derived from antigen‐specific IPSC in the same arthritic mouse model.^173^ Specific tissues or organ antigen targeting by IPSC‐derived CD4^+^ Tregs allowed a faster cell response on the inflammation site. They also showed that the adoptive transfer of autoantigen‐specific IPSC‐CD4^+^ Tregs significantly reduced the CD8^+^/CD4^+^ ratio in the pancreas of diabetic mice.^174^ Our own protocol of fully functional CD4^+^ and CD8^+^ Treg differentiation from human ES or IPS using FOXP3 transduction during the differentiation process allowed efficient generation of FOXP3^+^CD4^+^ and FOXP3^+^CD8^+^ Tregs (unpublished data). In addition, recently the knockdown of the polycomb group protein EZH1 was shown to activate lymphoid differentiation potential from pluripotent stem cells (PSCs) and this combined to FOXP3 transduction could improve Treg differentiation from PSCs.^175^

All these studies demonstrate the proof of concept that Tregs derived from ES cells or IPSC are an alternative source of T and Treg cells in immunotherapy.

## 7 ENGINEERING OF CD8^+^ TREGS

### 7.1 TCR/CAR and others

In recent years, many clinical trials have used polyclonal Tregs or expanded Tregs.^176^ However, polyclonal Tregs are non‐specific and can potentially cause global immunosuppression. Several studies have demonstrated that the use of antigen‐specific Tregs was much more effective than polyclonal Tregs in animal models.^7^, ^52^, ^177^, ^178^ Specific antigen Tregs can be generated by cultivating them with APCs containing specific antigens or using TCR engineering techniques. However, expansion with APCs can remain quite ineffective because of the few specific antigen Tregs present in the original polyclonal cells and difficulty to translate to the clinic. Therefore, TCR engineering appears as a promising technique to obtain antigen‐specific Tregs.^179^, ^180^, ^181^ Nevertheless, the use of TCRs remains restricted to a given MHC and TCR cannot be identified in all diseases, this is why another strategy providing antigen specificity emerged a few years ago with spectacular results in cancer: the CAR. The extracellular part of this synthetic receptor binds to surface molecules on the cells independently of the MHC, unlike TCRs, and independently of the patient's HLA haplotype since the specificity is provided by antibody sequences.^182^ The manufacture of CARs has evolved over the years, today there are four generations of CARs. The 1st generation contains a single chain variable fragment of an extracellular monoclonal antibody, a transmembrane domain and finally an intracellular domain CD3z to conduct the signal.^183^ For the second generation, a costimulation domain linked to CD3z has been added: CD28 (cytotoxic potential) or 4‐1BB (persistence and decrease exhaustion).^184^ The third generation contains the CD3z, CD28 and 4‐1BB domains which improve persistence and anti‐tumor activity.^185^ Finally, the fourth generation called T cell redirected for universal cytokine‐mediated killing (TRUCKs) for TRUCKs allows the production of inducible cytokines after the recognition of the molecule of interest by the CAR. This system could make it possible to recruit non‐CAR cells on the tumor site, for example through the secretion of chemokines to promote their elimination.^186^ Cells are now mainly used for cancer immunotherapy. CAR‐T cells targeting CD19 are effective in the treatment of malignant hematological diseases in preclinical and clinical trials.^182^, ^187^, ^188^ They were approved by the US food and drug administration for clinical treatments in 2017. This technology has shown its full potential in cancer therapy, it is now extended to therapies using Tregs. CAR Tregs' ability to control an inflamatory bowel disease in mice^189^ and in another model of colitis in mice^190^ has been demonstrated. Clinical studies show the use of Tregs to prevent GVHD after allogeneic hematopoietic stem cell transplantation.^191^, ^192^, ^193^ However, one study shows that the transfer of Tregs after hematopoietic stem cell transplantation can lead to overall immunosuppression and thus an increase in viral infections in the patient.^194^ This is why a more specific therapy is being sought and the use of antigen‐specific CAR Tregs is on the rise. HLA‐A2 has been shown to be a common antigen of incompatibility in transplantation with a prevalence of approximately 50% in European phenotype patients. Over the last 3 years, three groups have used anti‐HLA‐A2 antibody sequences to generate CARs and shown that anti‐HLA‐A2 CAR‐transduced CD4^+^ Tregs in xeno‐GVHD and skin grafts in NSG mice humanized with PBMCs had a higher suppressive potential than control CD4^+^ Tregs.^178^, ^195^, ^196^, ^197^ This year, our group has shown for the first time the proof of concept that CD8^+^CD45RC^low/‐^ Tregs expressing a CAR directed against HLA‐A2 are significantly more suppressive on CD4^+^ and CD8^+^ effector T cell in vitro than polyclonal Tregs and are able to inhibit graft rejection and GVHD more effectively than polyclonal Tregs in vivo in a model of HLA‐A2^+^ skin graft rejection and HLA‐A2^+^ PBMC GVHD in NSG mice (manuscript submitted). The transfer of antigen‐specific Tregs offers a promising strategy against pathologies characterized by aberrant immune activation such as transplant rejection and autoimmune diseases. In addition, the antigenic specificity provided by the CAR allows a much more targeted therapy and thus avoids the side effects of an overall immunosuppression of the patient.

In addition, insertion of a CNI resistance gene into Tregs is an interesting option considered for future clinical trials (the TacRes trial). Indeed, the stability of CD8^+^ Treg function may be optimized by genetic modifications. The CRISPR/Cas9 system opens new possibilities for stabilizing Foxp3 expression in Tregs.^198^ Besides, demethylation of Foxp1 could help Foxp3 DNA binding and increase CD8^+^ Treg suppressive function as reported for CD4^+^ Tregs.^199^ Otherwise, targeting genes such as HIF‐1alpha KO would switch Treg metabolism from glycolytic‐driven migration to an oxidative phosphorylation‐driven immunosuppression.^200^ On the other hand, overexpression of Hdr1 could help Tregs to face to ER stress response to an inflammatory environment.^201^ Further studies are required to assess the relevance of targeting these genes to improve the stability of CD8^+^ Tregs.

### 7.2 Safety system

To avoid any adverse event, genetic modifications to introduce a safety switch into the cells prior to the adoptive transfer have been developed. There are several techniques involving suicide genes. The two most commonly used techniques are Herpes Simplex virus thymidine kinase (HSV‐TK) and human inducible caspase 9 (iCasp9). HSV‐TK is the first suicide gene studied. The interaction of HSV‐TK with Ganciclovir leads to cell suicide by polymerase and DNA synthesis disruption.^202^ This technique has been tested in clinical phases I and II in France.^203^ While effective, this approach is limited by the immunogenicity of HSV‐TK expression with resultant rejection of modified cells.^204^ iCasp9 is very effective, 30 minutes after the injection of the molecule allowing his dimerization (CID) there is elimination of 90% of the T cells modified for iCasp9. The dimerization of iCasp9 with CID results in a cascade of caspases that is responsible for cell death by apoptosis.^204^, ^205^, ^206^, ^207^ However, this approach also has disadvantages such as the possible toxicity of the remaining cells and the possible autonomous dimerization of iCasp9 leading to the elimination of the cells before their action. An iCasp9 gene has been introduced into the CAR construct for effector T cell‐based therapy clinical trials in cancer (NCT03696784, NCT03016377, NCT03721068, NCT01822652).^208^

Other approaches such as controlling the intensity or toxicity of T or Treg cells by adding an “on switch” system via the CAR are being studied. The T cell response can be controlled by the recognition of two antigens simultaneously.^209^, ^210^, ^211^ Kloss et al showed in a mouse prostate cancer model that T cells co‐transduced with a CAR against two antigenic prostate tumors (prostate‐specific membrane antigen and prostate stem cell antigen) were able to destroy tumors expressing both antigens. However, these cells have no effect on cells with only one of the two antigens.^210^ Conversely, it is possible to transduce T cells with an inhibitory CTLA‐4‐ or PD‐1‐based iCAR to limit cytokine secretion, cytotoxicity, and proliferation.^212^ Overall, all this work provides an opportunity to specify selectivity and mimic the activity of modified T cells in order to reconcile processing power and safety.

## 8 CONCLUSION

Altogether, we believe that CD8^+^ Tregs are powerful players in immune tolerance that should be developed further through careful and systematic analyses in rodent models and human diseases. Although there is always some way to go, all these recent advances and upheavals will bring us to a better understanding of the CD8^+^ Tregs and the next generation CD8^+^ Tregs usable for cell therapy in transplantation and autoimmune diseases.
